# Thermoresponsive Microgel Coatings as Versatile Functional Compounds for Novel Cell Manipulation Tools

**DOI:** 10.3390/polym10060656

**Published:** 2018-06-12

**Authors:** Katja Uhlig, Thomas Wegener, Yvonne Hertle, Johannes Bookhold, Magnus Jaeger, Thomas Hellweg, Andreas Fery, Claus Duschl

**Affiliations:** 1 Branch Bioanalytics and Bioprocesses (IZI-BB), Fraunhofer-Institute for Cell Therapy and Immunology, 14476 Potsdam, Germany; magnus.jaeger@bfr.bund.de (M.J.); claus.duschl@izi-bb.fraunhofer.de (C.D.); 2 GeSiM mbH, 01454 Großerkmannsdorf, Germany; wegener@gesim.de; 3 Department of Physical and Biophysical Chemistry (PC III), Bielefeld University, 33615 Bielefeld, Germany; yvonne.hertle@uni-bielefeld.de (Y.H.); johannes.bookhold@uni-bielefeld.de (J.B.); thomas.hellweg@uni-bielefeld.de (T.H.); 4 Executive Office, German Federal Institute for Risk Assessment (BfR), 10589 Berlin, Germany; 5 Institute of Physical Chemistry and Polymer Physics, Leibniz Institute for Polymer Research Dresden, 01069 Dresden, Germany; fery@ipfdd.de; 6 Chair of Physical Chemistry of Polymeric Materials, Technical University Dresden, 01069 Dresden, Germany

**Keywords:** non-invasive cell detachment, cell cultivation, cell-based assay, stimuli-responsive polymers

## Abstract

For the effective use of live cells in biomedicine as in vitro test systems or in biotechnology, non-invasive cell processing and characterisation are key elements. Thermoresponsive polymer coatings have been demonstrated to be highly beneficial for controlling the interaction of adherent cells through their cultivation support. However, the widespread application of these coatings is hampered by limitations in their adaptability to different cell types and because the full range of applications has not yet been fully explored. In the work presented here, we address these issues by focusing on three different aspects. With regard to the first aspect, by using well-defined laminar flow in a microchannel, a highly controllable and reproducible shear force can be applied to adherent cells. Employing this tool, we demonstrate that cells can be non-invasively detached from a support using a defined shear flow. The second aspect relates to the recent development of simple methods for patterning thermoresponsive coatings. Here, we show how such patterned coatings can be used for improving the handling and reliability of a wound-healing assay. Two pattern geometries are tested using mouse fibroblasts and CHO cells. In terms of the third aspect, the adhesiveness of cells depends on the cell type. Standard thermoresponsive coatings are not functional for all types of cells. By coadsorbing charged nanoparticles and thermoresponsive microgels, it is demonstrated that the adhesion and detachment behaviour of cells on such coatings can be modulated.

## 1. Introduction

Adherent cells are an important class of cells. The in vitro processing, manipulation, and analysis of adherent cells are essential tasks in biomedical research, tissue engineering, toxicology, and biotechnology. Successful protocols not only depend on high levels of efficiency and precision but also on the preservation of the vitality of the cell material. In this context, the non-invasive detachment of adherent cells from synthetic cultivation substrates or scaffolds is crucial for making these cells available for cell-based approaches in the fields mentioned above.

Standard cell detachment by enzymes (e.g., trypsin) results in unspecific digestion of membrane proteins. These destroyed membrane proteins cannot be investigated after detachment (e.g., using patch clamp or FACS) until the cells have replaced them. In contrast, surface-mediated cell detachment by thermoresponsive polymers does not destroy membrane proteins, and is hence usually referred to as “non-invasive” in the literature [1].

Thermoresponsive polymer coatings have been identified as a valuable tool for achieving non-invasive control over cell adhesion [2,3,4,5,6]. Thermoresponsive polymers exhibit a sharp reversible structural transition upon temperature change around the lower critical solution temperature (LCST). Above the LCST the polymers show a compact conformation, whereas below the LCST the polymers swell in aqueous solutions and exhibit an open, highly-hydrated structure. During recent years, considerable progress has been achieved in exploiting the potential of such coatings for cell cultivation, tissue engineering, and the development of assays. Polymers of choice for cell cultivation applications include poly(*N*-isopropylacrylamide) (poly(NiPAm)), polyethylene glycol (PEG) and poly(vinyl methyl ether), as they show an LCST that is a few degrees below the physiological cultivation temperature, at between 26 and 33 °C [2,7,8,9]. When appropriately immobilised to solid substrates, thermoresponsive polymer coatings mediate protein and cell adhesion at 37 °C. Decreasing the temperature below the LCST triggers non-invasive cell detachment due to polymer hydration, which reduces protein and cell adhesion. The use of such coatings may replace common detachment methods that rely on proteases which impair the vitality of cells and invalidate results obtained from them.

Recently, we have introduced thermoresponsive coatings that are based on microgels. Besides excellent functional properties such as changing hydration, elasticity, and topography depending on temperature (strongly influencing cell adhesion), these coatings possess some features that greatly expand their applicability [10]. As the size of the microgels is large enough to support firm attachment to many substrate materials, various simple formation methods can be employed for the production of versatile and inexpensive thermoresponsive coatings. Dipping, spin coating, spraying, spotting, and printing all allow the fabrication of highly functional coatings. The latter two methods facilitate local application of the microgels in order to produce defined patterns and structures [11]. In all cases, the microgels form highly regular monolayers. In addition, adhesion and detachment of cells to the coatings can be easily modulated by introducing microparticles with distinct surface properties into the coatings.

In the following, we present results that highlight the functionality and versatility of thermoresponsive microgel coatings. As an example for demonstrating the ease of integrating these coatings into microfluidic systems, we systematically investigate the detachment of cells as a function of the flow velocity of the medium. The potential of the patterned microgel coating for the development of cell assays is demonstrated in two examples. Finally, we report results on the modulation of the cell adhesion properties by the addition of microparticles into the thermoresponsive microgel coatings.

## 2. Materials and Methods

Polymer synthesis: The microgel (MZ140) was synthesized through precipitation polymerization as previously described [10]. In short, *N*-isopropylacrylamide (NIPAM; Sigma-Aldrich 97%, Saint Louis, MO, USA) was cross linked by *N*,*N*’-methylenebis(acrylamide) (BIS; Sigma-Aldrich, 99%) to obtain thermoresponsive microgels. For this purpose, 10.568 mmol NIPAM and 0.98 mmol BIS were dissolved in 150 mL purified water. The solution was heated up to 70 °C under continuous stirring and purged with nitrogen. Subsequently, the reaction was initiated as described previously, and the work-up of the obtained product was performed as in our previous works. The obtained microgels were characterized by PCS [11].

Sample preparation: For uniform coating with thermoresponsive microgels, freshly cleaned glass substrates (20 × 20 mm^2^, Menzel, Braunschweig, Germany) were covered with 200 µL of 1% poly(ethylenimine) solution (PEI, Sigma Aldrich, Steinheim, Germany) for one minute. The supernatant was removed using a spin coater (CPS 20, Semitec, Dresden, Germany) for 20 s with 3000 rpm. Afterwards, 200 µL of a 0.5 wt % microgel suspension was incubated for 30 s on the PEI-modified glass substrate. Finally, the supernatant was again spun for 10 s at 2000 rpm and for 10 s at 3000 rpm.

To alter the surface properties of thermoresponsive microgel coatings, carboxylated polystyrene beads (Fluoresbrite^®^ YG Carboxylate (0.20 µm), Polysciences Europe GmbH, Hirschberg an der Bergstrasse, Germany) were mixed with the microgel suspension before the surface immobilization process mentioned above. To this end, a 0.6 wt % microgel suspension and a 0.6 wt % polystyrene bead (PS beads) suspension were prepared and both were mixed according to the following volume ratios: 1:0; 200:1; 100:1; 50:1; 5:1, and 0:1. To simplify the nomenclature of the various mixing ratios, we herein use a concentration indication in percent which is related to mixing volumes. It should be noted that the densities of microgel and PS beads are different, and thus the percentages are rough approximations. Thus, 0% is equivalent a ratio that did not contain PS beads (1:0), and the percentages of 0.5%, 1%, 2%, 20%, and 100% are equivalent to ratios of 200:1, 100:1, 50:1, 5:1, and 0:1, respectively. For visualisation of the fluorescent PS bead distribution on the surface, a confocal laser scanning microscope (510 Meta, Zeiss, Oberkochen, Germany) equipped with an argon laser and a 63 × / 1.4 oil immersion objective was employed. For image acquisition, the pinhole was set to 1 Airy unit (image slice of approximately 0.7 µm).

For generating patterned coatings on cyclo olefin polymer substrates (COP ibiTreat, ibidi, Planegg, Germany), a nano-plotter (NP2.1, GeSiM, Großerkmannsdorf, Germany) equipped with a piezo dispenser (Nano-Tip A, GeSiM, Germany) was employed. Circular spots with individual volumes of 300 pL were dispensed in a grid of 353 µm. The overall area of spots was 1 cm^2^. To generate lines, the spotting grid was decreased in one dimension to 190 µm and individual spots were dispended using a suspension concentration of 0.35 wt %. During the spotting process, the liquid was completely evaporated. Afterwards, new spots were placed between the previously positioned spots to generate lines, resulting in a final spotting distance of 95 µm. The other dimension was increased to establish a line distance of 600 µm.

Cell culture: L929 mouse fibroblasts (ACC 2, DSMZ, Germany) were cultivated in DMEM containing HEPES (25 mM), FCS (10%), penicillin–streptomycin (1%), and L-glutamine (2 mM, all Biochrom, Germany). CHO-K1 cells (ACC 110, DSMZ Germany) were cultivated in Ham’s F12 supplemented with FCS (10%) and penicillin– streptomycin (1%, all Biochrom, Berlin, Germany) at 37 °C and 5% CO_2_.

Shear force assay: The microsystems were incubated with cell medium overnight. During this time, air bubbles occurred, and were flushed out of the system with additional medium. Then, 2 × 10^6^ L929 mouse fibroblasts mL^−1^ were injected via the side channel and cultivated without additional medium supply for one day in an incubator. After 30 min under microscopic observation at ~22 °C, flow rates of up to 2000 µL min^−1^ were applied using a 1-mL glass syringe (ILS, Stützerbach, Germany) operated by a syringe pump (SP230IWZ, WPI, Hitchin, UK). The average flow velocity across the channel cross section was calculated by dividing the volume flux (volume supplied by the syringe pump per time) by the channel cross sectional area (A = 0.045 mm^2^). In order to exclusively measure the effect of the physical shear force and to avoid any influence from biological or chemical changes (shear history), the cells should be exposed to every velocity step as quickly as possible. For practical reasons (the syringe pump being operated manually), a shear time of 10 s was selected. Thus, every 10 s, the flow velocity was increased stepwise until all cells were rinsed off the surface or the maximum pump velocity was reached.

Quantification of the shear force: The hydrodynamic shear force *F* acting on a spherical cell in contact with the channel bottom was numerically derived using the program Comsol Multiphysics 4.3a for any of the chosen flow velocities according to our estimations in previously published work [12].

Cell migration assay: For the cell migration assay, two kinds of patterns were used. The substrates coated with microgel spots were placed in petri dishes and 3 × 10^4^ CHO-K1 cells cm^−2^ were seeded. The COP substrates coated with microgel lines were stuck in microfluidic channels (Sticky-Slide IV 0.4, ibidi, Germany) and 2.5 × 10^4^ L929 cells were seeded in the microchannel. After one day of cell culture at 37 °C, the samples were cooled to 22 °C for 30 min. Afterwards, the cells located on the microgel were rinsed off in a petri dish using a 1-mL Eppendorf pipette and in the microchannels with a 10-mL syringe. All cell migration observations were performed with a fully automated set-up (Cell-R, Olympus, Hamburg, Germany) equipped with a 10 × / 0.3 objective and an incubation chamber (Air Conditioning Unit, Evotec, Hamburg, Germany).

Cell adhesion assay: To observe the cell adhesion on the substrates coated with microgel and PS beads, the samples were placed in a six-well plate and 2 × 10^4^ L929 cells cm^−2^ were seeded in each well. Immediately after seeding, the samples were placed under the microscope at 37 °C for recording a time lapse film. The delay until the time lapse acquisition started was approximately five minutes. The percentage of cells which changed their morphology from a round to a spread state over one hour was analysed. Subsequently, cell detachment from the surfaces upon temperature decrease was investigated. To this end, the samples were cooled to 22 °C for 30 min after one day of cell culture. Then, the percentage of cells which reduced the cell surface contact area from a spread to a round state was determined. Finally, the samples were rinsed using a 1-mL Eppendorf pipette.

## 3. Results and Discussion

### 3.1. Shear Force Assay

**Figure 1:**
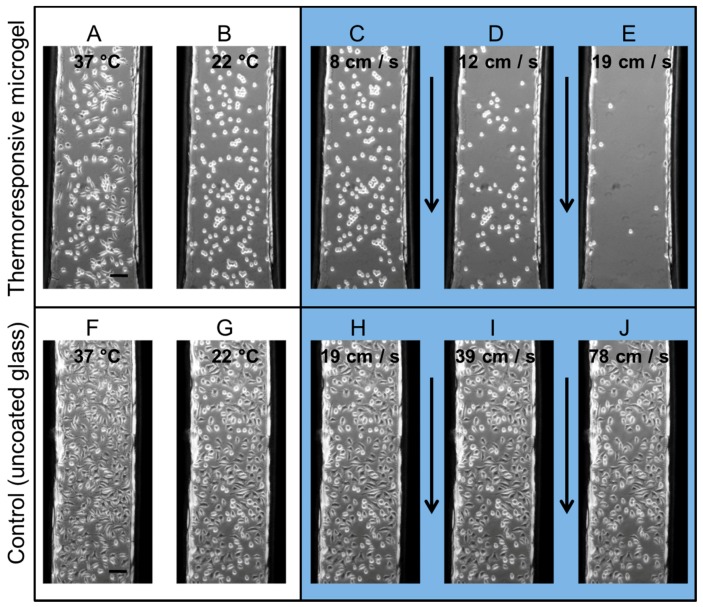
Phase contrast images of L929 mouse fibroblasts cultivated in a microchannel on a homogeneously microgel-coated glass substrate (**A**–**E**) and on an uncoated glass substrate (control) (**F**–**J**). The cells were cultivated one day in the microchannel at 37 °C (**A**,**F**). After having been exposed to room temperature (~22 °C) for 30 min (**B**,**G**), the microchannels were flushed using flows of stepwise increasing velocity (duration of 10 s for each step, the yellow arrow indicates the flow direction). The remaining cells in the microchannel at different flow velocities are shown at (**C**) 8 cm s^−1^, (**D**) 12 cm s^−1^, and (**E**) 19 cm s^−1^ on a microgel-coated substrate, and (**H**) 19 cm s^−1^, (**I**) 39 cm s^−1^, and (**J**) 79 cm s^−1^ on a glass substrate. Despite the high flow rates in the uncoated channels, the cells did not detach, although they were easily removed from the polymer coatings at low fluid velocities. Scale bars: 100 μm.

To quantify the shear force required to detach individual cells from the microgel in their cell-repellent state, we used microfluidics as a tool for reproducibly generating well-defined flow conditions. In these, the bottom of the microchannel was formed by homogeneous microgel coatings or, as a control, plain glass substrates. L929 mouse fibroblasts were cultivated for one day at 37 °C in these microchannels. The cells adhered and spread on the thermoresponsive polymers. Then, the whole setup was cooled to 22 °C under microscopic observation (Figure 1A,B,F,G). The fibroblasts changed their morphology on the thermoresponsive microgel coating from a spread to a round state and remained in a spread state on the control surface without the thermoresponsive polymer. Subsequently, a defined flow of stepwise increasing velocity was applied to the microsystem and the number of remaining cells in the microchannel was detected at each velocity (Figure 1C–E,H–J). At a flow rate of 8 cm s^−1^, the cells were still unaffected by the flow. With higher flow rates, the cells started to detach and the cell number in the channel decreased: at 12 cm s^−1^ and 19 cm s^−1^ approximately 50% and 10% of the initial cell number remained, respectively. At the same flow rate of 19 cm s^−1^ there was no cell detachment of cells growing on the control glass substrates. When the flow velocity was doubled and quadrupled, 98% and 88% of cells, respectively, still remained in the microchannels on the non-coated glass bottom.

**Figure 2:**
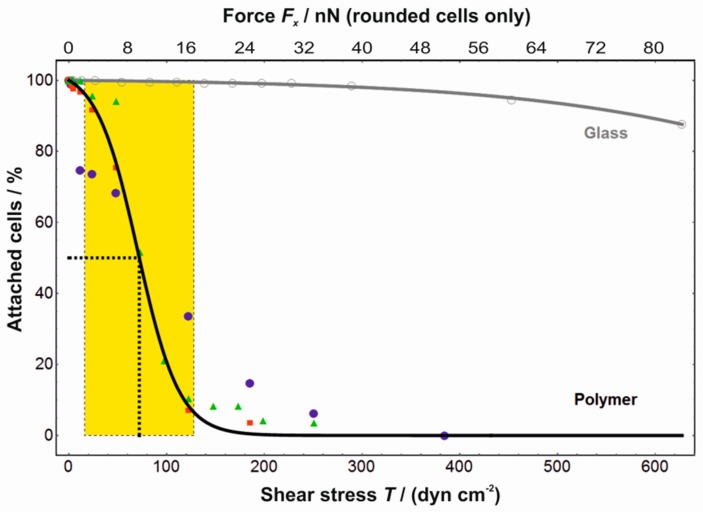
Quantification of the force required to detach individual L929 cells from the microgel coating in a cell-repellent state using a microfluidic approach. The fraction of remaining cells in the microchannel is a function of the shear stress *T* (lower abscissa) and of the shear force *F_x_* acting on individual cells with spherical morphology (upper abscissa). Note that consequently the force axis is only valid for the rounded cells on the microgel coatings, since the spherical shape assumed in the numerical calculation does not hold for the spread morphology on glass. Filled symbols denote experiments using the microgel coating (*n* = 3), open symbols indicate control experiments on glass. All data sets were individually fitted with a logistic function (sigmoid curve), each yielding two parameters: the shear stress, where 50% of the cells remained (*F*_50_), and the width of the transition (*w_F_*). For the thermoresponsive microgel coating (black curve), the weighted averages were *T*_50_ = (72 ± 1) dyn cm^−2^ and *w_T_* = (56 ± 3) dyn cm^−2^, while on glass (grey curve) a complete cell detachment could not be achieved because of setup limitations. The shear stress needed to detach cells from glass was at least one order of magnitude higher than from the microgel coating. Fifty percent of the cells were detached at an average force of (7.9 ± 0.1) nN with a transition width of (6.6 ± 0.4) nN.

Approximating the rounded cells as spheres of radius 10 µm for which the perimeters rest on the channel bottom, we numerically estimated the force *F_x_* acting on them parallel to the flow direction and the shear rate at their position (Comsol Multiphysics). From the latter, the shear stress *T* was derived by multiplying with the viscosity (0.9 mPa s). In the calculations, the pressure gradient was adjusted so as to obtain the required average flow velocity across the channel. Three individual sets of experiments and a control are combined in the diagram shown in Figure 2. The average shear stress *T*_50_ needed to detach 50% of cells and the width *w_T_* of the transition were calculated, where *w_T_* is the interval between the two maxima of the second derivative (curvature) of the fitted function. For a 50% cell detachment from microgel coatings, a shear stress of (72 ± 1) dyn cm^−2^ was needed with a transition width *w_T_* of (56 ± 3) dyn cm^−2^. The shear stress needed to detach cells from glass was at least one order of magnitude higher in comparison to the stress needed on microgel coatings (Figure 2). Assuming a spherical shape for the cells on the microgel coating (after cooling), this shear stress *T*_50_ corresponded to a force of *F*_50_ = (7.9 ± 0.1) nN.

The force needed to detach a single cell can be expected to increase with the extent of the focal adhesion area, i.e., with the sum of cell surface interactions. The broad width of the transition on glass indicates a considerable heterogeneity of the cell adhesion, probably depending e.g., on the cell cycle or migratory activity. It should be noted that the aim of our flow experiments was the quantification of the required shear force for single-cell detachment. Therefore, a short period of only 10 s per flow velocity was chosen to avoid the shear history exerting an adverse influence on the cell detachment rather than the actual cell surface interactions [13]. For future research, we will continue the shear experiments by using a parallel flow setup and by changing the channel geometry to create shear gradients.

### 3.2. Cell Migration Assay

**Figure 3:**
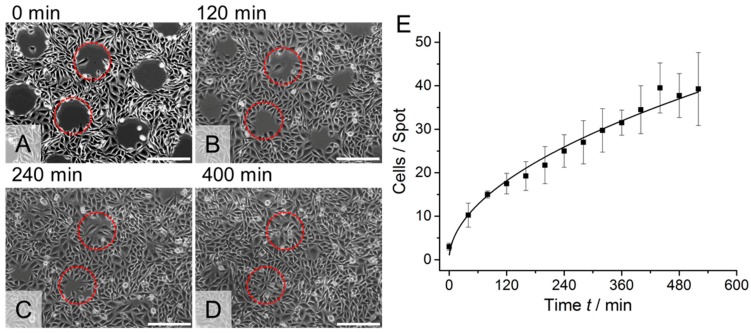
Representative images of time lapse microscopy of CHO-K1 epithelial cells on patterned thermoresponsive microgel substrates. (**A**) The cell patterning was achieved by detachment of cells from the microgel spots after the sample was incubated for 30 min below the lower critical solution temperature (LCST) at 22 °C. Cells attached on the uncoated areas remained on the substrate. When the temperature was increased to 37 °C, the cells started to migrate to the now cell-free regions (**B**,**C**) and formed a closed cell monolayer after 400 min (**D**). Two representative microgel coated areas per picture are indicated by red circles. Scale bars: 200 μm. (**E**) Quantification of the cell migration onto the microgel spots at 37 °C as a function of time. (**A**) adapted with permission from [11]. Copyright 2016 American Chemical Society.

**Figure 4:**
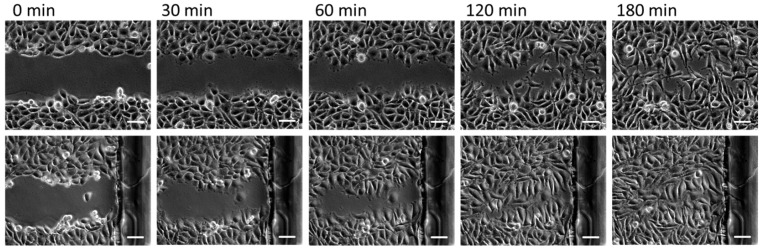
Representative images of time lapse microscopy of L929 mouse fibroblasts on thermoresponsive microgel lines in a microchannel (two different positions, first row and second row). The cell patterning was achieved by detachment of cells from the microgel lines after the sample was incubated for 30 min below the LCST at 22 °C. Cells attached on the uncoated areas remained on the substrate (first column). When the temperature was increased to 37 °C, the cells started to migrate to the now cell-free regions and formed a closed cell monolayer after 180 min (last column). Scale bars: 100 μm.

Another remarkable feature of our thermoresponsive polymer system is the ease with which it is possible to pattern the polymers to enable local cell detachment, e.g., for performing cell migration assays. For this purpose, we deposited picoliter volumes of microgel suspensions onto a substrate using an inkjet spotter. We positioned the different polymer suspensions separately in a grid to obtain single spots. Furthermore, we changed the spotting protocol to generate continuous lines of microgel coating by letting the spots overlap each other. The substrate with microgel spots was placed in a petri dish and the substrate with lines was assembled into a commercially available microfluidic channel. Both samples were cultivated for one night with either CHO-K1 epithelial cells (spots) or L929 mouse fibroblasts (lines). Then, the temperature was reduced to 22 °C for 30 min. The cell–polymer interaction changed due to the temperature reduction and enabled a defined detachment of cells located on the polymer coating by rinsing with a pipette (Figure 3) or flushing with a syringe (Figure 4). As a result, the cells on the polymer patterns could be removed locally. On the uncoated regions, the cells remained on the surface. After temperature increase to 37 °C, the cells started to resettle the polymer regions because the polymer became cell-attractive again. The CHO-K1 cells and mouse fibroblasts had again formed a confluent layer after 400 and 180 min, respectively.

### 3.3. Cell Adhesion Assay

**Figure 5:**
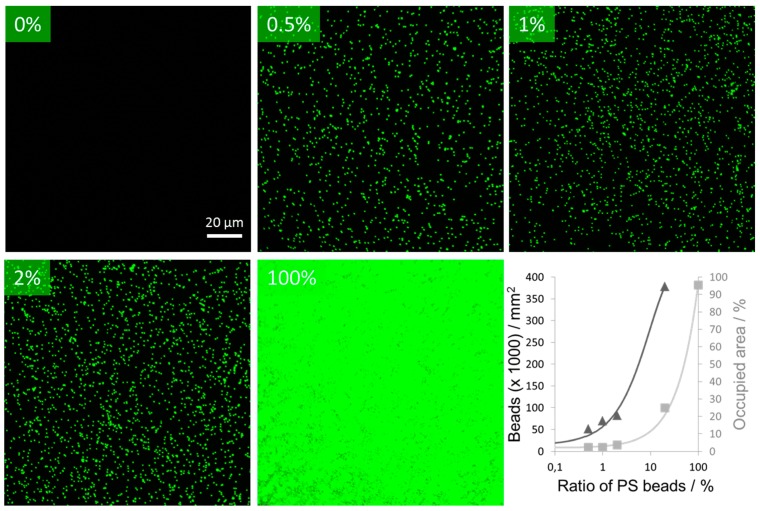
The surface density of polystyrene (PS) beads coadsorped with microgels can predictably and homogenously be adjusted through the bead ratio of the coating suspension. Fluorescence microscopy images of poly(ethylenimine) (PEI) glass slides taken after coadsorption of microgels and fluorescent PS beads using various mixing ratios of the suspensions (0%, 0.5%, 1%, 2%, and 100% beads). With increasing numbers of PS beads in the solution, the number of beads on the surface also increased. The number of beads per area was counted (triangles, left ordinate). The area occupied by the fluorescent beads was measured (squares, right ordinate). Both measures are approximately linearly related to the ratio of beads in the coating suspension (note logarithmic abscissa). Solid lines added only as guide to the eye.

**Figure 6:**
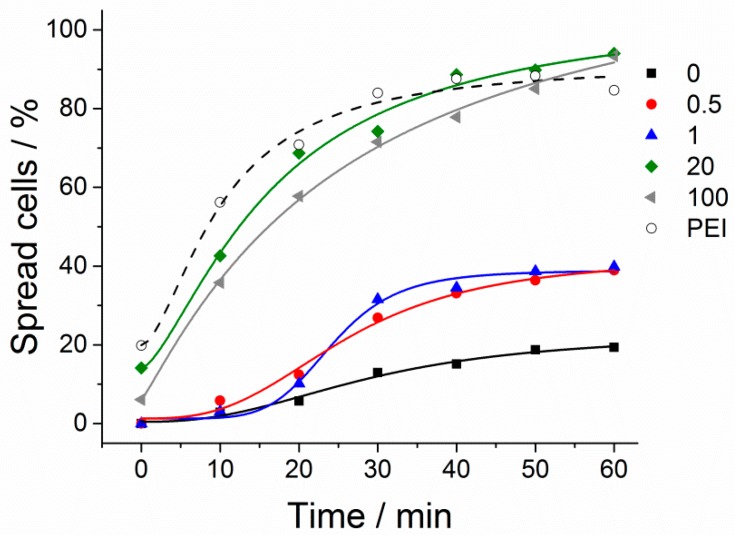
Quantification of the cell spreading of *n* number of cells on glass substrates coated with different ratios of microgel and PS beads (0% beads, black, *n* = 155; 0.5% beads, red, *n* = 154; 1% beads, blue, *n* = 168; 20% beads, green, *n* = 120; 100% beads, grey, *n* = 89) and a PEI-modified glass substrate (open circles).

The cell behaviour on the thermoresponsive polymer coatings can be further improved by integrating additional surface properties [14,15,16,17]. Direct modification of the thermoresponsive microgels is one option to manipulate cell surface interactions. For this purpose, the microgels can either be coupled with functional groups to enhance (e.g., RGD moieties) or reduce (e.g., PEG moieties) the cell attachment. However, such modifications may decrease the performance of the thermoresponsive polymers due to unfavourable molecular interaction between the two moieties [18]. To avoid such molecular interferences of polymer modifications, our approach is to decouple the microgel from secondary properties by mixing the microgel suspension with bead suspensions before the surface preparation. The advantage of using particles is a defined spatial separation of the thermoresponsive polymers and additional cell adhesion-affecting polymers. To verify our approach, we used carboxylated polystyrene beads (PS beads) with a diameter of 200 nm and mixed them in various ratios with thermoresponsive microgels. These PS beads were fluorescently labelled to allow an analysis of the PS bead distribution on the surface. After coadsorption of the microgels and PS beads, we observed homogeneously distributed fluorescence spots over the whole surface, indicating a homogeneous bead distribution (Figure 5). As expected, the amount of carboxylated PS beads adhered on the surface increased linearly with an increasing amount of PS beads in the coating suspension. To test the impact of the integrated PS beads on the cell adhesion, we analysed the time cells needed to spread on such coatings. To this end, L929 mouse fibroblasts were seeded on substrates produced with different mixing ratios of microgels and beads. The cell morphology was investigated and quantified by using an optical microscope (Figure 6). Initially, the cells were in a round state due to the lack of cell surface interaction immediately after seeding. On surfaces made purely of thermoresponsive microgels without PS beads, 19% of cells changed their morphology from a round to an elongated shape within one hour. When small amounts of PS beads (1% or 0.5%) were incorporated into the thermoresponsive microgel layer, the cell attachment was already significantly improved: after one hour, 40% and 38% of the cells were elongated and spread on these coatings. On pure PS bead-modified surfaces and thermoresponsive substrates mixed with 20% PS beads, cells adhered in a manner similar to that on the PEI-modified substrates, showing a delay of cell spreading time of five to ten minutes. After one hour, 84% to 94% of the cells had spread on all three different surfaces (on PEI, 84%, on PS beads, 85%, and on substrates mixed with 20% PS beads, 94%). On the control substrate modified with polyethylenimine, the cells adhered and spread quickly. On PEI coatings, the cells started to spread during the time between seeding and data acquisition (19%), i.e., within about five minutes.

**Figure 7:**
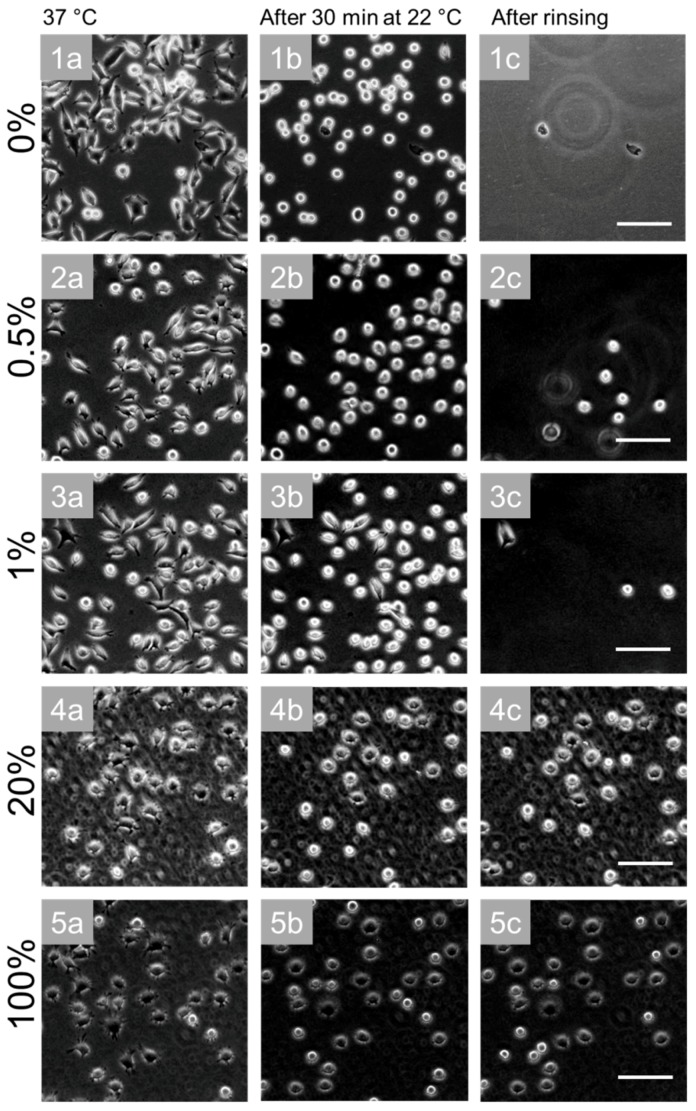
Cell assays to investigate cell detachment on microgel coatings doped with PS beads upon temperature decrease below the LCST. Phase contrast images of L929 mouse fibroblasts cultivated on glass substrates prepared from solutions of different ratios of microgel and PS beads: (**1a**–**1c**) 0% PS beads, (**2a**–**2c**) 0.5% PS beads, (**3a**–**3c**) 1% PS beads, (**4a**–**4c**) 20% PS beads and (**5a**–**5c**) 100% PS beads. After one day, the cells were spreading at 37 °C on all substrates (**a**). After having been exposed to room temperature (~22 °C) for 30 min, the cells reduced the cell surface contact area (**b**). With increasing PS bead concentration, the cell morphology change became less pronounced. After rinsing, nearly all cells were detached from substrates using 0%, 0.5%, and 1% PS beads (**1**, **2**, **3**). On surfaces prepared with 20% (**4**) and 100% PS beads (**5**), nearly all cells remained on the substrate. Scale bars: 100 μm.

To test whether the functionality of the thermoresponsive microgels to detach cells upon temperature decrease below the LCST was still preserved in bead-doped coatings, we performed dedicated cell detachment assays with the surfaces described previously. After one day of culture at 37 °C, the cells were elongated and spread on all surfaces (Figure 7a). After temperature reduction to 22 °C for 30 min, the cells decreased their adhesion area depending on the PS bead density on the surface (Figure 7b). On pure microgel coatings without PS beads, 90% of cells changed their morphology to a round shape. On surfaces made of microgel suspensions containing 0.5% PS beads, 84% of the cells became rounded (with 1% → 68%, 20% → 36%, and 100% → 26%). These observations indicate that the functionality of the thermoresponsive microgel coatings was already impaired by small amounts of PS beads. Nevertheless, the cell detachment after surface rinsing was efficient for surfaces using up to 1% PS beads. The advantage in better cell spreading gained through addition of PS beads to microgel suspensions clearly outweighed the reduced cell detachment resulting from the less pronounced morphology change.

Our coadsorption experiments prove that the cell surface interaction can easily be tuned by integrating additional properties. We did not employ additional, e.g., chemical modifications of the bead surfaces, but they would most probably have a substantial impact on cell adhesion. Another improvement of cell surface interaction may be attributed to overall size or the size ratio of microgel and beads. Currently, the diameter of the colloidal microgels is about 600 nm below and 300 nm above the LCST. The cross section of the PS beads used here was 200 nm. A size optimisation could reduce the impact of the beads on the cell detachment ability of the microgel.

## 4. Conclusions

Thermoresponsive microgel coatings allow the non-invasive detachment of cells from cultivation substrates. In an effort to qualify these coatings for a wider range of applications, we addressed a number of issues that still required improvement in order to meet this goal. We showed that the laminar flow in a microfluidic channel could be used to non-invasively detach cells from a support using well-defined shear conditions. As an example for demonstrating the potential of patterned thermoresponsive coatings for the improvement of cell assays, we established a simple wound-healing assay. Finally, by coadsorbing charged nanoparticles and thermoresponsive microgels at various compositions, it was possible to tune the properties of thermoresponsive coatings. This strategy should allow the optimisation of the adhesion and detachment behaviour of coatings for specific cell types. In addition, this approach avoids the problem that arises when molecular entities are directly bound onto the thermoresponsive polymers, as chemical modifications of these polymers usually impair their functionality. We believe that thermoresponsive cell culture equipment based on microgels will soon be a helpful tool for many protocols, as thermoresponsive coatings show a high level of functionality as well as various interesting features, and their production is simple and inexpensive.

## Abbreviations

FACS	fluorescence-activated cell sorting
BIS	*N*,*N*′-methylenebis(acrylamide)
PCS	photon correlation spectroscopy
DMEM	Dulbecco’s Modified Eagle Medium
HEPES	4-(2-hydroxyethyl)-1-piperazineethanesulfonic acid
FCS	fetal calf serum
COP	cyclo-olefin polymer
RGD	arginylglycylaspartic acid (Arg-Gly-Asp)
